# An Unusual Case of Cecal Perforation: Accidental Ingestion of a Tooth in an Elderly Trauma Patient

**DOI:** 10.7759/cureus.45467

**Published:** 2023-09-18

**Authors:** Shamon Gumbs, Gonzalo Ausqui, Tonny Orach, Alexius Ramcharan, Brian Donaldson

**Affiliations:** 1 Department of Surgery, Columbia University College of Physicians and Surgeons, Harlem Hospital Center, New York, USA

**Keywords:** critical care, blunt trauma, swallowed foreign body, cecal perforation, trauma

## Abstract

Foreign body ingestion is a common occurrence in the United States, with the majority passing asymptomatically. In cases where complications occur, such as intestinal perforation, it may present as an acute abdomen with diagnostic challenges regarding the etiology. A 70-year-old male was brought to the emergency department (ED) after he jumped from the second floor of a burning building, sustaining 10% second-degree burns to his forearms. He was intubated for concerns of inhalational injury and resuscitated. His intensive care unit (ICU) course included the management of respiratory failure, sepsis, and acute kidney injury. On hospital day 28, imaging showed moderate pneumoperitoneum with ascites. He was taken for abdominal exploration, during which it was noted that there was gross fecal contamination and a 1 cm cecal perforation. After resection of the ileocecum, it was left in discontinuity due to hemodynamic instability and contamination. He was brought for a second-look laparotomy in 48 hours, and an incisor tooth was found in the right pelvis, and an ileocolic (ileum-ascending colon) anastomosis was performed. His post-operative course was complicated by an anastomotic leak and an intra-abdominal collection. Despite attempts at source control with percutaneous drainage, the patient remained septic with a poor prognosis. Goals of care were discussed, and the decision was made to de-escalate care. Although there is literature on foreign body ingestion resulting in intestinal perforation, there is a paucity of literature highlighting the importance of dental exams in elderly trauma patients, the incidence of perforation due to tooth ingestion, and maintaining a high index of suspicion for an acute abdomen in unusual presentations of sepsis.

## Introduction

Foreign body (FB) ingestion is a common occurrence in the United States, with the majority of FB passing asymptomatically through the gastrointestinal tract. In cases where complications occur, such as intestinal perforation, it may present as an acute abdomen with diagnostic challenges regarding the etiology. CT imaging of the abdomen is often obtained due to its high sensitivity in evaluating intestinal perforation. The most common sites of perforation due to FB ingestion occur at areas of physiological angulation or narrowing (such as the ileocecal valve) or previous surgery. Outcomes depend upon early diagnosis and rapid treatment [1,2]. This case was presented as a poster abstract at the ASCRS Annual Scientific Meeting 2023 in Seattle, Washington, USA.

## Case presentation

We present a case of a 70-year-old male brought into the ED with level 2 trauma after he jumped from the second floor of a burning building. He sustained second-degree partial-thickness burns to his forearms, approximately 10% of his total body surface area, with some soot around his nose. He was intubated for suspicion of inhalational injury and resuscitated in the trauma bay. He had whole-body CT scans, which did not identify any other acute injuries. He was admitted to the burn intensive care unit (ICU), and an inhalational injury was ruled out via a bronchoscopy. The patient’s ICU course included management of acute respiratory failure, sepsis (from pneumonia and other undiagnosed sources at the time), and acute kidney injury. The patient was being managed on broad-spectrum antimicrobials.

**Figure 1:**
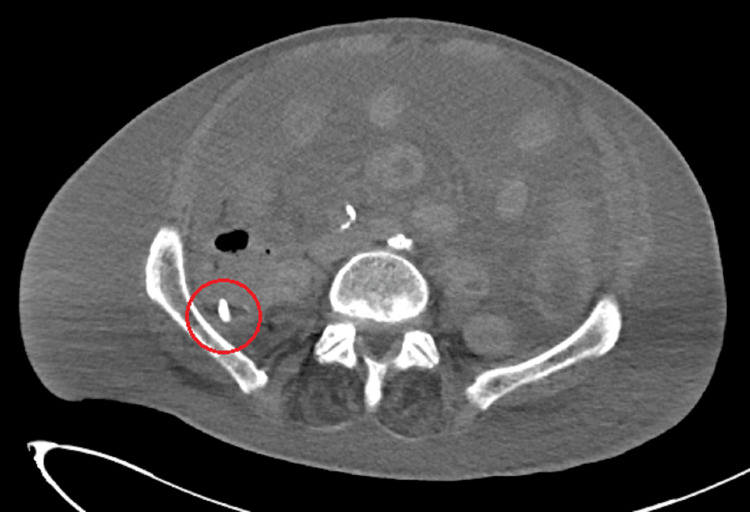
Axial view CT abdomen/pelvis showing ascites and a hyperdensity in the right pelvis concerning for a foreign body.

On hospital day 28, the patient developed leukocytosis, became oliguric, and had a distended, tense, abdomen with grimacing during abdominal palpation. A CT abdomen/pelvis without IV contrast was obtained, which showed a new mild to moderate pneumoperitoneum with a large amount of ascites and diffuse anasarca (Figure 1) when compared to his initial CT on arrival.

**Figure 2:**
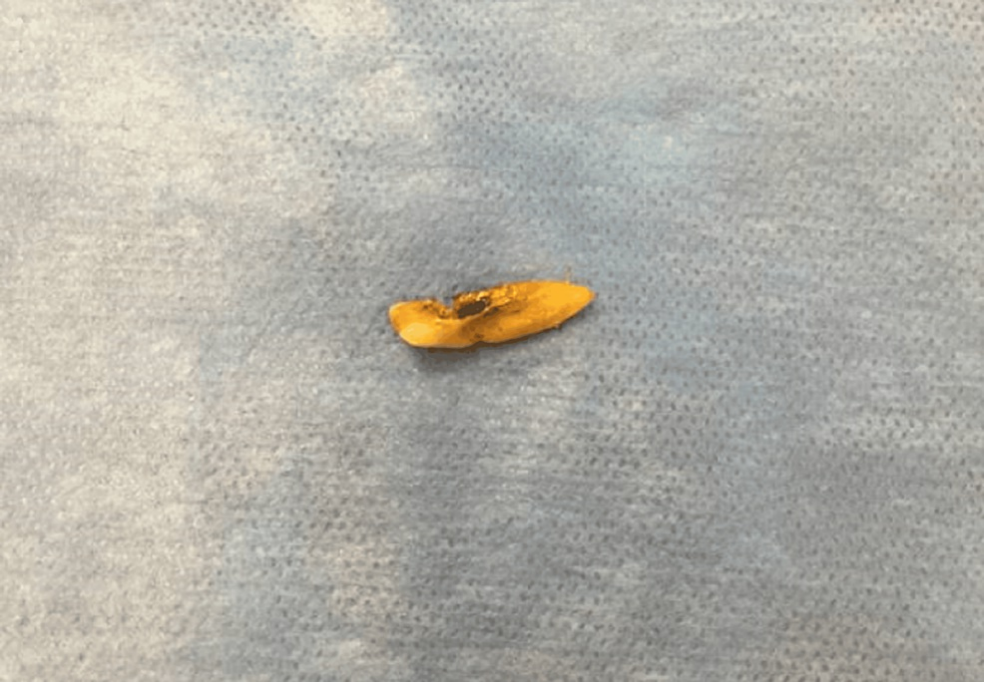
An elongated incisor tooth was discovered intra-peritoneally.

The patient was taken to the operating room for abdominal exploration. Intraoperatively, there was noted to be gross fecal contamination, with extensive peritoneal fluid appearing bilious. An approximately 1 cm cecal perforation was discovered on the antimesenteric border. Given the patient's hemodynamic instability and gross contamination, the ileocecum (approximately 5 cm of the ileum) was resected and left in discontinuity. The peritoneal cavity was extensively washed out. The patient was then sent to the ICU for continued resuscitation. The patient was taken back in 48 hours for a second-look laparotomy. During the surgery, a loose incisor tooth was discovered in the right pelvis (Figure 2).

Given the improved hemodynamic status and adequate source control, the decision was made to create a stapled ileocolic (ileum to the ascending colon) anastomosis, and the abdomen was closed. The patient remained in the ICU being managed for sepsis on broad-spectrum antibiotics (such as meropenem, linezolid, and caspofungin).

On the post-operative day (POD) 26, the patient still had persistent leukocytosis (ranging from 14-15 x 10^9^/L) on broad-spectrum antibiotics. A repeat CT scan of the abdomen and pelvis was obtained, which showed a large rim-enhancing collection in the right abdomen, concerning an intra-abdominal collection. As well as a dependent hyperattenuating component suspicious for extravasated intraluminal contrast concerning ileocolic anastomotic leakage. The patient was made nothing by mouth and started on parenteral nutrition, as he was previously started on nasogastric tube feeds on POD 1 after creating the anastomosis. Interventional radiology was consulted and performed ultrasound-guided percutaneous drainage of the right abdominal collection and pigtail catheter insertion for continued aspiration. The aspirate was 500 ml of purulent green fluid from the right abdomen and a clear yellow aspirate from the left lower quadrant. The cultures were positive for *Candida albicans*, *Candida auris*, and *Candida glabrata*; the patient was placed on contact isolation and continued on antifungals. Despite attempts at additional source control, the patient remained septic with a poor prognosis. Goals of care discussion were held with the family, and the decision was made to transition to comfort care as opposed to undergoing additional invasive surgeries to achieve source control. The patient was then pronounced dead later that day.

## Discussion

GI perforation is associated with significant morbidity and mortality, primarily due to the secondary bacterial peritonitis that can develop. GI perforation involves the loss of the bowel wall's integrity and the subsequent release of intraluminal contents into the sterile peritoneal cavity. The intestines can be perforated due to various mechanisms, including foreign body penetration, extrinsic or intrinsic obstruction, infection, ischemia, or loss of wall integrity without foreign body perforation [1]. Our paper will focus on GI perforation due to foreign body penetration.

Foreign body (FB) ingestion is a common occurrence in the United States. The majority of reported foreign body ingestions occur in children and are usually accidental. However, in the adult population, it is often due to intentional ingestion, likely as a result of psychiatric disorders, intoxication, or secondary gain. In some cases, it can be accidental due to, but not limited to, dentures (causing defective tactile sensation) or sensory deficits due to cerebrovascular accidents [2,3]. FB ingested can be blunt or sharp; blunt objects pass through the GI tract without incident; sharper objects are more likely to lead to complications such as GI perforations. Perforations can occur at any location in the GI tract, but more often at areas of physiological angulation or narrowing (such as the ileocecal valve), congenital malformations, or previous surgery [2,4]. Some of the most commonly accidentally ingested FB include fish bones, chicken bones, and toothpicks, which often cause perforation due to being sharp and elongated. The incidence of the type of FB ingested varies based on the dietary habits of the population involved. In the literature, most of the perforations reported were due to dental prostheses and other ingested foreign bodies. Through our literature review, there was no other identifiable publication on perforation as a result of a tooth. This is why this paper is important to spread awareness and maintain a high index of suspicion, especially in the elderly or maxillofacial trauma populations. 

Patients may not be able to report ingesting FB, which can delay diagnosis and treatment. Oftentimes, the ingested FB is not detected on plain radiography due to it not being radiopaque and being obscured by intestinal gas. For instance, a prospective study by Ngan of 358 patients with fish bone ingestion revealed that plain radiography had a sensitivity of only 32%. Patients then present later with intestinal perforation and symptoms of an acute abdomen [2,5].

In the workup of patients with an acute abdomen, CT imaging of the abdomen and pelvis plays an important role in evaluating intestinal perforation due to its high sensitivity [2]. It has a reported diagnostic sensitivity of 95-98%, a specificity of 95.1-97%, and an accuracy of 95.6%. CT imaging findings that can aid in identifying regions of GI perforation include the thickened intestinal segment, localized pneumoperitoneum, regional fatty infiltration, associated intestinal obstruction, or any combination of these findings. It is important to note that none of these findings are specific [6].

In our case, the patient was managed in the ICU, initially intubated and sedated, then transitioned to a tracheostomy. However, the patient remained non-verbal. Intestinal perforation was not initially suspected due to the unusual presentation. The patient’s abdomen was noted to become increasingly distended, associated with reduced urine output and leukocytosis. It was only after obtaining a CT of the abdomen that free intraperitoneal air was noted, and the patient was taken to the operating room for surgical exploration.

Once a diagnosis of intestinal perforation or peritonitis has been made, management involves an exploratory laparotomy. Some authors have described utilizing laparoscopy or laparoscopic-assisted techniques. The treatment can include simple primary suture repair of the defect and resection of the bowel (with or without primary anastomosis) [3]. The decision to perform primary anastomosis should be considered cautiously in cases of gross purulent or feculent peritonitis or hemodynamic instability due to the increased incidence of anastomotic breakdown. 

Patients with GI perforations develop systemic inflammatory response syndrome (SIRS) or sepsis and can have hemodynamic instability and the “triad of death” (coagulopathy, hypothermia, and acidosis). The challenge in these cases is to be able to provide life-saving surgery without causing additional physiologic compromise from the stress of surgery. Utilizing damage control techniques has been described in the literature, where the main goal is obtaining source control as quickly as possible without prolonging operative time to restore functional anatomy. Oftentimes, the bowel is left in discontinuity to resect diseased or necrotic bowel with plans for re-evaluation in 24-28 hours to re-establish functional anatomy. In between the operative room, the patient was managed in the ICU for continued resuscitation [1].

## Conclusions

FB ingestion is a common occurrence in the United States, with the majority of FB passing asymptomatically through the GI tract. In cases where complications occur, such as intestinal perforation, it may present as an acute abdomen with diagnostic challenges regarding the etiology. In the literature, most of the perforations reported in the elderly population were due to dental prostheses and other ingested foreign bodies. Through our literature review, there was no other identifiable publication on perforation as a result of a tooth. This is why this case is important to spread awareness and maintain a high index of suspicion, especially in the elderly or maxillofacial trauma populations. Clinicians should maintain a high index of suspicion for foreign body ingestion as a cause of unusual presentations of intestinal perforations because a preoperative history of FB ingestion is rarely obtained. ​​Outcomes depend upon early diagnosis and rapid treatment, including early source control.
